# Interfacing low-energy SAW nebulization with Liquid Chromatography-Mass Spectrometry for the analysis of biological samples

**DOI:** 10.1038/srep09736

**Published:** 2015-05-15

**Authors:** Karina Tveen-Jensen, Frank Gesellchen, Rab Wilson, Corinne M. Spickett, Jonathan M. Cooper, Andrew R. Pitt

**Affiliations:** 1 Division of Biomedical Engineering, University of Glasgow, Oakfield Avenue, Glasgow, UK. G12 8LT; 2 School of Life and Health Sciences, Aston University, Aston Triangle, Birmingham, UK. B4 7ET

## Abstract

Soft ionization methods for the introduction of labile biomolecules into a mass spectrometer are of fundamental importance to biomolecular analysis. Previously, electrospray ionization (ESI) and matrix assisted laser desorption-ionization (MALDI) have been the main ionization methods used. Surface acoustic wave nebulization (SAWN) is a new technique that has been demonstrated to deposit less energy into ions upon ion formation and transfer for detection than other methods for sample introduction into a mass spectrometer (MS). Here we report the optimization and use of SAWN as a nebulization technique for the introduction of samples from a low flow of liquid, and the interfacing of SAWN with liquid chromatographic separation (LC) for the analysis of a protein digest. This demonstrates that SAWN can be a viable, low-energy alternative to ESI for the LC-MS analysis of proteomic samples.

Soft ionization techniques for the mass spectrometry (MS) of biomolecules, first developed more than two decades ago, have revolutionized the field of biological mass spectrometry and opened up the entire field of proteomics^1^,^2^,^3^. Electrospray ionization (ESI) and matrix-assisted laser desorption and ionization (MALDI) currently form the cornerstones of mass spectrometric analysis of proteins, peptides and polar metabolites, as both generate ions from labile biomolecules under relatively low energy conditions that preserve the molecular integrity of the sample^4^,^5^,^6^. In ESI, an additional advantage is that it is compatible with analytes in solution. A solution of the analyte is passed through a capillary held at a high electric potential, causing highly charged droplets to emerge from the needle tip. Evaporation of the solvent then leads to the formation of ionized molecules in the gas phase. Therefore, a significant advantage of ESI is that it can straightforwardly be coupled online to liquid chromatography (LC), combining the power of chromatographic separation with high-resolution mass detection and molecular identification in a mass spectrometer (LC/MS)^7^.

Although ESI has been widely adopted, and is used in the majority of biological mass spectrometry studies, it has some shortcomings. For instance, ESI has been shown to induce electrochemical oxidation in samples, even under conditions where no visible corona discharge occurs^8^, and there is still a need for ionization methods that deposit less energy into ions upon ion formation and transfer for detection for the analysis of more labile molecules^9^,^10^, such as phosphopeptides^11^,^12^, peptides rich in serine^13^, proteins^10^,^14^ and especially non-covalent assemblies of molecules such as protein complexed with ligand or other proteins^15^,^16^,^17^,^18^ and self-assembled inorganic supramolecular complexes^19^. Hence, the identification of alternative low-energy ionization methods is needed either for specialized applications or to address the shortcoming of ESI, and this has led to the development of cold-spray^20^ and other sources^21^. For these methods to be generally useful it is highly advantageous if they can be interfaced with liquid flow methods, especially with liquid chromatography which has become a fundamental and integral part of MS workflows for proteomics and metabolomics.

Recently, it has been shown that Surface Acoustic Wave-based Nebulization (SAWN) is capable of transferring non-volatile biological samples from solution to the gas-phase for subsequent analysis by MS^22^,^23^. Surface acoustic wave (SAW) technology has been employed for many years in a variety of microfluidic devices^24^ and biosensors^25^. However, the application of SAW as a nebulization technique is a relatively recent development of this technology^23^,^26^,^27^, and remains to be fully exploited, especially for sample introduction in mass spectrometry. It has also been demonstrated that SAWN is a particularly low energy method for analyte introduction from the liquid phase into a mass spectrometer^28^, suggesting that it has some of the key attributes to complement ESI. These studies also demonstrate that SAWN is compatible with a number of different MS sources from different manufacturers. The ionization process between SAWN and ESI is clearly different, with no direct potential or ground being applied to the sample, and this may also be responsible for differences observed. The ionization process of SAWN is not fully understood. The surface of 128° rotated Y cut LiNbO_3_ used in the transducer chip forms strong dipoles and associated with this is an inherent negative charge^29^ but due to the piezoelectric nature the polarity can change in the presence of a mechanical wave. The surface charge present at the surface can be increased by the application of an external ac field applied to an interdigitated transducer. The sinusoidal excitation induces a mechanical wave, which as the wave traverses the piezoelectric material also induces a reciprocal process and charge flow or current in the material occurs. This excess charge might then be transferred to liquid on nebulization^23^,^30^.

We have previously shown that SAWN of peptide samples can be interfaced with MS in continuous mode or for the analysis of individual droplets^23^, but these studies were limited to simple samples and fixed solvent systems. We report here the use of a simple flow interface for sample introduction via SAWN, examine the effects of flow and concentration on MS data quality, and show for the first time that SAWN can be effectively interfaced with liquid chromatography with gradient elution for the analysis of mixtures by MS.

## Results

In order to demonstrate that SAWN-MS is capable of detecting peptides of different physicochemical properties and concentration in mixtures in flow mode, we analyzed a mixture of Glu-1 Fibrinopeptide B (GluFib, EGVNDNEEGFFSAR), an easily ionizable and generally multiply-charged peptide and ALILTLVS, a more hydrophobic and generally singly-charged peptide, at a 50:1 μM concentration respectively in infusion mode at 5 μLmin^-1^. Both peptides were detected without difficulty, and their identity was easily confirmed by MSMS (Supplementary fig. 1).

**Figure 1:**
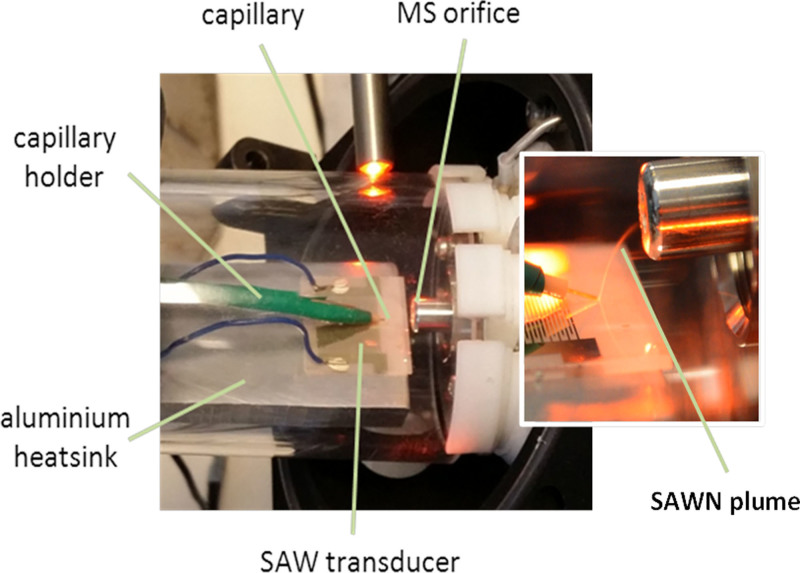
Annotated photographs of the SAWN apparatus. Insert shows plume generated from SAWN nebulization of flow during liquid chromatography.

For optimum performance it was found that the tip of the capillary should be placed so that it was just in contact with the surface of the transducer, and its position on the transducer finely adjusted until a “sweet spot” was found that gave rise to a fine plume of droplets. The use of an xyz stage with fine adjustment to position the capillary tip made this optimization significantly easier. It was also beneficial to make some fine adjustment to the frequency and amplitude of the signal applied to the transducer during initial setup to maximize the performance of the system, especially when changing flow rates. The position of the transducer relative to the mass spectrometer was adjusted so that the plume of droplets impacted just away from the orifice of the mass spectrometer (fig. 1). Once the interface had been optimized, the system remained stable for many hours.

**Figure 2:**
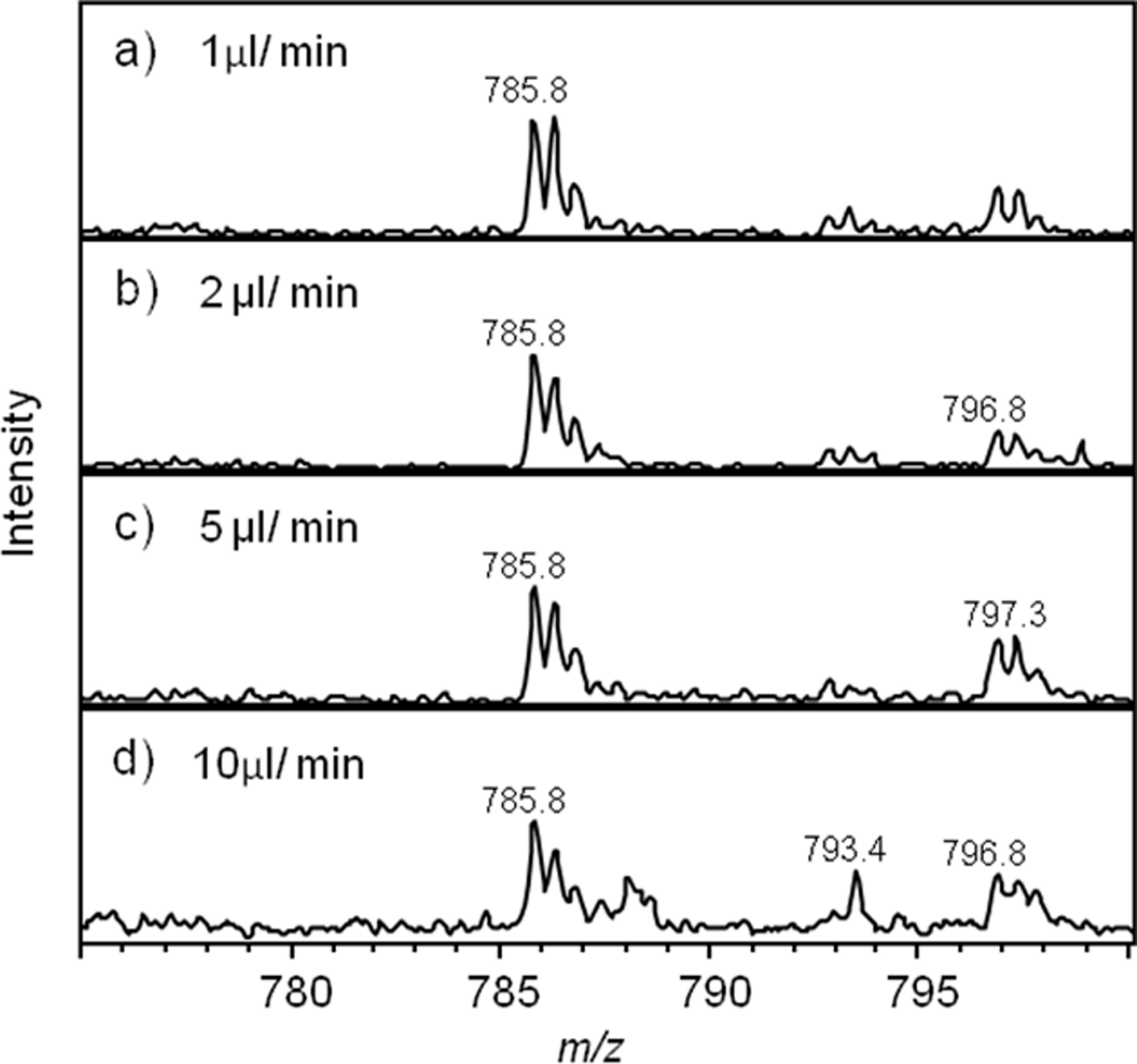
Effect of flow rate on data quality. 100 μM [Glu]-1 fibrinogen B, *m/z* 785.8 (doubly charged ion) in 50% acetonitrile, 0.1% formic acid in water was infused into the mass spectrometer via the SAWN interface. Data was averaged over 0.5 min. Flow rates were: (a) 1 μLmin^-1^, (b) 2 μLmin^-1^, (c) 5 μLmin^-1^, (d) 10 μLmin^-1^. As flow rate increases above 5 μLmin^-1^ the signal to noise decreases and additional peaks become apparent in the spectra. Signal to noise ratios for the different flow rates are shown in table 1.

In order to assess the effect of flow rate on the MS signal generated from SAWN, a solution of 100 μM GluFib was infused at a range of flow rates from 0.5–30 μLmin^−1^. Reliably obtaining stable signal from flow rates below 1 μLmin^−1^ was problematic, as plume formation often became intermittent. Above 1 μLmin^−1^ the system performance was good up to 30 μLmin^−1^ (the highest flow rate tested). However, as the flow rate was increased above 5 μLmin^−1^ significant background signal became apparent in the spectra and the signal to noise ratio decreased (fig. 2 and table 1), probably due to incomplete desolvation of the nebulized droplets. Therefore, the optimum flow rate range for our system was 1–5 μLmin^−1^, which is ideally suited for interfacing with capillary flow LC separation.

**Figure 3:**
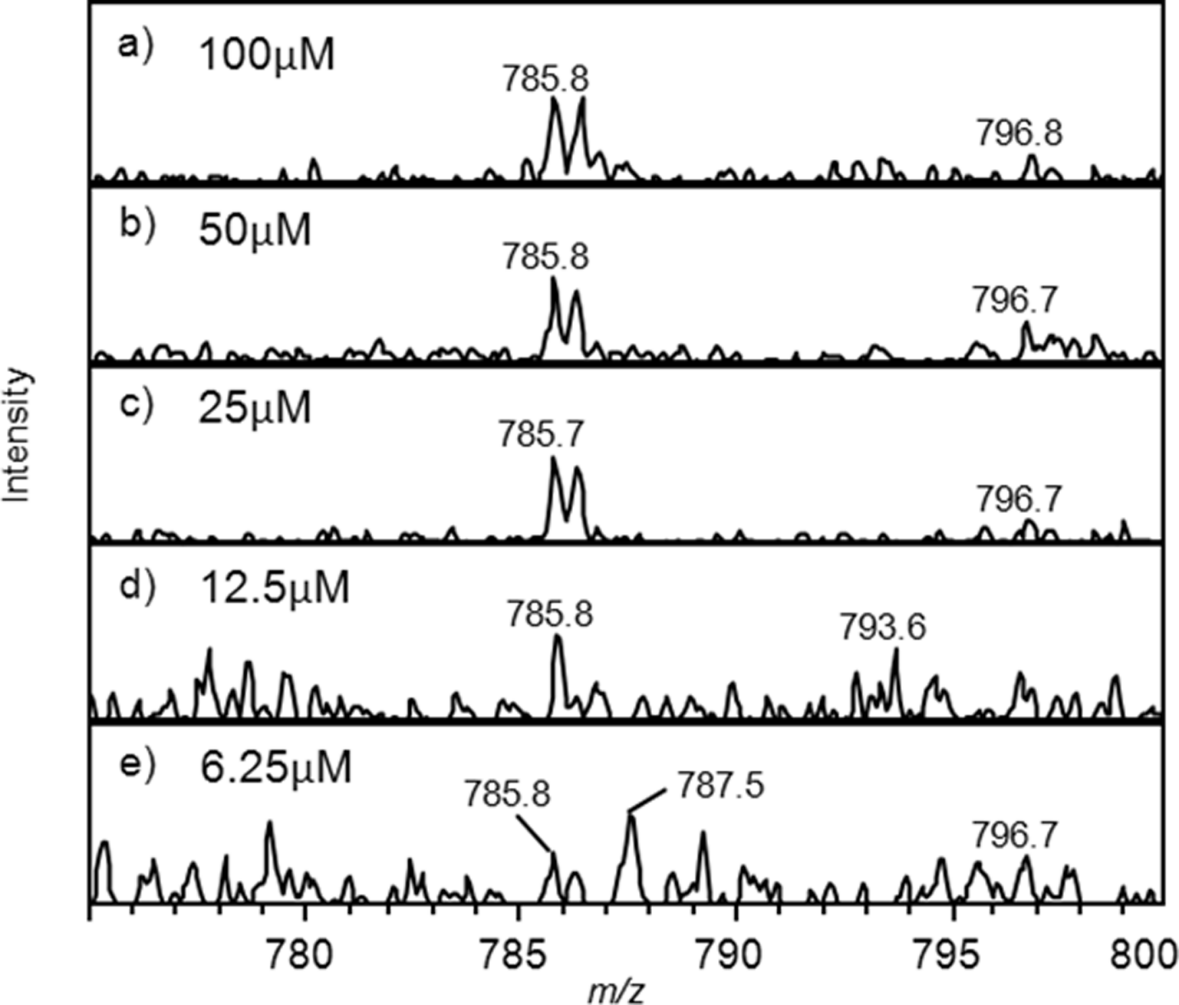
The effect concentration on the signal to noise ratio. [Glu]-1 fibrinogen B, *m/z* 785.8 (doubly charged ion) in 50% acetonitrile, 0.1% formic acid in water was infused into the mass spectrometer via the SAWN interface. Data was averaged over 0.5 min. Concentrations (and amount of sample consumed) were: (a) 100 μM (20 pmoles) (b) 50 μM (10 pmoles) (c) 25 μM (5 pmoles) (d) 12.5 μM (2.5 pmoles) and (e) 6.25 μM (1.25 pmoles). Signal to noise ratios for the different concentrations are shown in table 1. S/n decreases markedly below 12.5 μM, and was <3 for 6.25 μM.

The general sensitivity of this system was assessed by determining the limit of detection for a GluFib solution in MS mode with concentrations of 6.25 to 100 μM infused at 2 μLmin^−1^ (fig. 3 and table 1). Signals were integrated across a 0.5 minute window, and a good signal for MS data was obtained down to 25 μM GluFib, corresponding to 10 pmoles of sample consumed. MSMS data could be obtained down to 6.25 μM (1.25 pmoles sample consumed).

We then interfaced the flow-based SAWN nebulizer with an LC system and tested its use for the on-line separation and identification of peptides from a tryptic digest of human serum albumin (HSA), which gives rise to a mixture of peptides and is commonly used as a standard in proteomic analysis. 30 pmol and 3 pmol of the digest were loaded and separated on a 300 micron capillary reversed-phase column at a flow rate of 2 μLmin^−1^, a combination frequently used for the analysis of biomolecules. Data was extracted using the Bruker DataAnalysis software and analyzed using Bruker Biotools and the Mascot search engine to assess the quality of the data obtained. Full data for the peptide analysis is provided in Supplementary table 1. For the 30 pmol sample HSA was identified with very high statistical confidence (protein score 783), which is a measure of the quality of the data generated, and with good sequence coverage (45%), demonstrating that the nebulization/ionization is efficient for a range of peptides of different physicochemical properties. (Supplementary fig. 2). Even at 3 pmol (approximately 200 ng) of sample loaded onto the column, the data was of very good quality (protein score 433, sequence coverage 42%) (Supplementary fig. 2).

For comparison the same sample was analyzed using a standard electrospray ionization (ESI) source with the same column, LC system and mass spectrometer, loading 3 pmoles of the digest onto column, and running replicates to asses reproducibility. A Mascot search of the data demonstrated similar performance for SAWN and ESI. Protein identification scores of 399 +/− 143 and 538 +/− 32 were obtained for SAWN and ESI respectively, and sequence coverage gave a similar relationship with coverage of 39 +/− 9% and 52 +/− 6% respectively (errors are standard deviation, n = 4). These data suggest that the SAWN interface gives a similar quality of performance to the ESI source, although it is worth noting that the SAWN data is significantly more variable. The data was searched for oxidative modifications to assess whether there was any significant decrease in in-source oxidation for the SAWN source. Only methionine oxidations were identified for both sources. Quantitative analysis of the mass spectrometric data to assess the relative amount of oxidation of methionine, an amino acid residue that is particularly sensitive to oxidation, showed that levels were low (<10%) for both ionization methods. A slight decrease in oxidation was generally apparent in the SAWN sample, but the difference was not statistically significant, probably due to the low overall level of oxidative modification observed.

## Discussion

**Figure 4:**
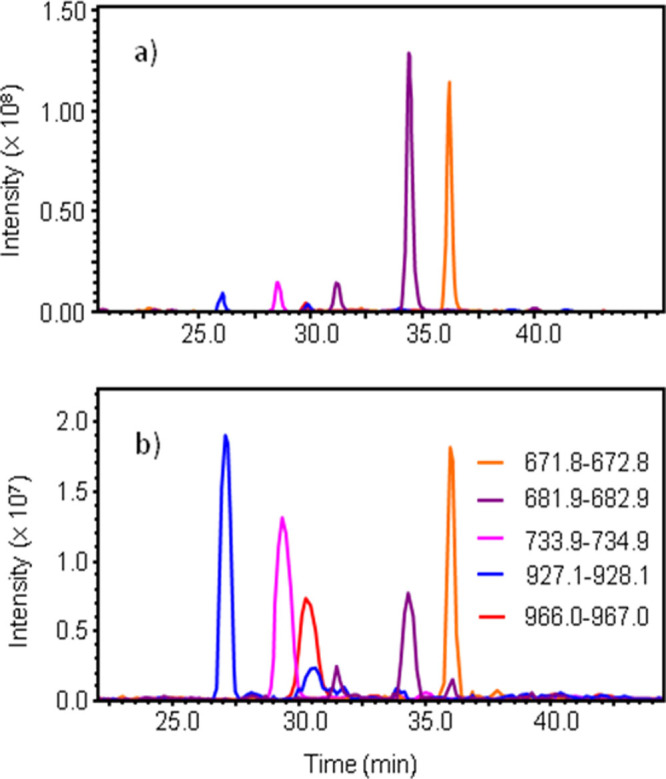
Comparison of extracted ion chromatograms (XICs) of selected peptides from a tryptic digest of human serum albumin from (a) electrospray ionization, and (b) SAWN.Like colours correspond to the same peptide in each analysis. Insert in (b) shows the legend for peptide *m/z* values.

The main reasons that ESI has been widely adopted as an MS ionization technique is that it is able to ionize and introduce into the gas phase labile, polar, non-volatile molecules, such as proteins and peptides, at relatively low energy, thus maintaining the integrity of these biomolecules, and that it can easily be coupled online to liquid chromatography to harness the high resolution separation power of this technique to analyze complex mixtures of molecules. The SAWN interface has been previously demonstrated to produce lower energy ionization than ESI, but to be most useful in the laboratory it must also be able to be integrated with LC analysis, including running gradients of aqueous and organic buffers, and efficiently ionize labile, polar biomolecules. While ultimately this is not as sensitive as the best ESI systems, it is still suitable for proteomic analysis, as demonstrated below. This is the first report of the use of proteomic methods for the analysis of a digested protein using LC separation coupled directly to SAWN. An interesting observation is that the peptide signal intensities appear more uniform across the whole chromatographic run for SAWN than with ESI, with the earlier-eluting, more hydrophilic peptides showing signal intensities comparable to the later-running peptides, suggesting less discrimination in the ionization (fig. 4). This may be due to a lower charge density on the droplets from SAWN leading to differences in desolvation and ionization mechanisms between the two methods^31^, or a more uniform nebulization/ionization. The use of the SAWN interface caused a very slight broadening of peak shape compared to the ESI source, but still gave very well resolved LC data, as demonstrated by the extracted ion chromatograms generated for some of the identified peptides (fig. 4). The broadening may be due to some additional mixing at the capillary tip due to the effect of the SAW vibration on the liquid in the tip, which is in contact with the chip surface, or in the liquid on the surface of the chip before nebulization. The results from the analysis of the data using the MASCOT statistical search engine suggests that SAWN performs reasonably against ESI, both for sequence coverage (number of peptides identified) and overall protein score, which is an indicator of depth and quality of data. It is noticeable that the SAWN data is more variable that the ESI data, which is an indicator that improvements need to be made in reproducibility, such as in chip manufacture.

The SAWN interface offers a viable alternative to ESI for biological sample introduction into a mass spectrometer, and it can be easily coupled to flow systems, and is entirely compatible with LC separation of samples. The source is most effective with low capillary flow rates, and is well matched with LC methods widely used for biomolecule analysis. We have demonstrated that the SAWN interface can be used for the analysis of proteomic style samples, using an LC separated tryptic digest of low pmole quantities of HSA, and the data has been shown to be comparable in statistical robustness to that obtained with ESI.

## Methods

### Reagents and materials

Sequencing grade porcine pancreatic trypsin was purchased from Promega. Human serum albumin (HSA) and human [Glu1]-Fibrinopeptide B (GluFib) were purchased from Sigma (Poole, Dorset). All other solvents and chemicals were of analytical quality and obtained from Fisher. Three inch LiNbO_3_ wafers were supplied by Roditi International Corp, the wafers were diced by ICT Engineering Technology; the transducer's interdigitated electrodes were fabricated using standard optical lithography and metallization via e-beam evaporation and lift off. Each electrode was 217 μm in width and 1.5 cm in length with spacing between each electrode equal to the electrode width. Each transducer had 20 electrodes pairs.

### Apparatus

All experiments were carried out using a HCT Ultra ion trap Mass Spectrometer running the Compass instrument control software (Bruker Daltonics, Bremen), in positive ion mode. The SAW nebulization apparatus consisted of an arbitrary signal generator (TTi 50 MHz) and a mini circuits RF amplifier (ZHL-5W-1) driving a LiNbO_3_ interdigitated transducer (IDT) at a range of resonant frequencies, as described previously^23^. The nebulizer was interfaced with the mass spectrometer using a modification of the standard MS interface; the general setup is shown in fig. 1. The MS interface used a standard spray shield on the end of the transfer capillary, which was held at −6 kV relative to ground. To minimize the effects of air currents in the laboratory the nebulization apparatus was placed in a 20 cm long 6 cm diameter Perspex tube mounted onto the front of the mass spectrometer. The transducer was mounted on an aluminium block heat sink in order to minimize heating effects, and positioned relative to the mass spectrometer aperture with the aid of a vertical height adjustable stage.

Sample introduction employed a polymer coated glass capillary with an inner diameter of 20 μm threaded through a fine solid tube held in a clamp mounted on a micrometer adjustable xyz stage. The clamp allowed for the positioning and the angle of the glass capillary relative to the transducer aperture while the xyz stage allowed for the positioning of the transducer relative to the MS orifice, in order to optimize signal. Sample introduction flow was generated by an Ultimate 3000 LC system (Thermo Fisher, Hemel Hempstead, UK) fitted with a capillary flow splitter controlled through Chromeleon (Thermo Fisher, Hemel Hempstead, UK) for standalone mode and the MS system using the Hystar software (v3.2 SR1, Bruker Daltonics, Bremen, Germany) for on-line LC analysis. Sample was introduced either by infusion using loop injection or liquid chromatographic separation as described below. Transducer frequencies of around 5 to 12 MHz and input signals to the amp of −6 to −4 dBm were used providing output powers of approximately 2 to 4 W. The power and frequency were optimized by fine adjustment to provide a stable plume when nebulizing for each transducer used and flow rate employed. The positioning of the transducer relative to the orifice was also adjusted depending on flow rate to give maximum signal. The parameter settings on the mass spectrometer were as follows: source temperature 365° C, drying gas 2 Lmin^−1^, nebulizer gas off, capillary potential −6 kV. Data were collected in positive ion enhanced scan mode. Other settings were optimized to give maximum signal intensity.

### Trypic digestion of Human Serum Albumin

Human serum albumin (1 mg/ml) was digested with trypsin in-solution as described in Mouls *et al.*^32^.

### Concentration and flow rate dependence of SAWN

To test the effect of flow rate 100 μM [Glu1]-Fibrinopeptide B (GluFib) in 50% aqueous methanol, 0.1% formic acid was delivered at flow rates ranging from 0.5–20 μLmin^−1^. To test the effect of sample concentration, solutions of GluFib in 50% aqueous methanol, 0.1% formic acid ranging from 100 μM to 6.25 μM were delivered at 2 μLmin^−1^. Data was averaged over 0.5 minutes and s/n was calculated as signal intensity against 5σ noise using the DataAnalysis software (Bruker Daltonics, Bremen, Germany).

### LC-MS SAWN analysis

The tryptic digest of HSA was resuspended in 2% acetonitrile, 0.1% formic acid in water prior to LC-MS analysis. Separation of the peptides was achieved by capillary flow reverse-phase chromatography using a C18 PepMap™, 5 μm, 5 mm × 0.3 mm i.d. trap column and an Acclaim PepMap™ 100, 300 μm × 15 cm, 3 μm, 100 Å, Nanoviper® C18 separation column (Thermo Fisher, Hemel Hempstead, UK). Samples were loaded onto the trap and washed for 4 min with 2% aqueous acetonitrile (0.1% formic acid) at 30 μLmin^−1^. The trap was then switched in line with the column and the peptides separated at 2 μLmin^−1^ using a gradient elution from 2% to 40% aqueous acetonitrile, 0.1% formic acid over 40 min, followed by a washing step at 90% aqueous acetonitrile, 0.1% formic acid for 4 min and re-equilibration with 2% aqueous acetonitrile, 0.1% formic acid for 10 minutes.

MS profile scan peptide data were collected in standard enhanced positive ion mode from 400 to 1500 *m/z*, averaging 5 spectra, with ICC control (accumulation of either 200,000 charges or for 200 ms), with active exclusion of the precursor ion after 2 spectra and release after 0.5 min. Collision induced dissociation (CID) fragmentation was performed on the three most intense ions within *m/z* 300–1,200, with the threshold for precursor ion selection set at an absolute intensity of 20,000. Singly charged ions were excluded. MSMS spectra were scanned from *m/z* 100–3,000 Da in enhanced mode averaging 5 spectra.

### Statistical analysis of peptide data

Raw data analysis was performed and extracted ion chromatograms generated using DataAnalysis software (v4.0 Build 234, Bruker Daltonik GmbH, Bremen, Germany). MS data was processed in Data Analysis using default parameters to generate a .xml table, which was then further analyzed using Biotools (v3.0, Bruker Daltonics, Bremen, Germany). Data was searched against the SwissProt database (13 September 2013) mammalian taxonomy using Mascot v2.4 (Matrix Science, London, UK) on an in-house server, selecting the variable modifications carbamidomethyl cysteine and methionine oxidation. The peptide tolerance was set at 0.8 Da and the fragment tolerance at 0.5 Da, choosing +2, +3 and +4 charge states and allowing for one missed cleave by trypsin.

## Author Contributions

A.R.P. and J.M.C. conceived the work and A.R.P., J.M.C. and C.M.S. were responsible for overall direction of the work. R.W., F.G. and K.T.J. collected the data. A.R.P., R.W., F.G., C.M.S. and K.T.J. were responsible for data analysis. The manuscript was compiled by A.R.P., C.M.S. and F.G. through contributions of all authors. All authors have given approval to the final version of the manuscript.

## Supplementary Material

Supplementary Information Supplementary figures 1 and 2 and table 1

## Figures and Tables

**Table 1:** Signal to noise (s/n) measurements for flow and concentration experiments

Flow rate (μLmin^-1^)	s/n^a^	SD	Concentration (μM)	s/n^a^	SD
10	24.3	12.1	100	24.3	2.9
5	32.3	12.4	50	30.6	3.5
2	32.5	11.6	25	29.1	3.4
1	33.9	11.5	12.5	7.6	1.9
			6.25	<3	n/d

^a^s/n is measured for noise at 5σ against peak intensity using the DataAnalysis software (Bruker Daltonics, Bremen, Germany). Measurements are made on spectra averaged over 0.5 min, and are averages of 8 measurements for flow rate and 4 measurements for concentration. Errors are given as standard deviation (SD). n/d – unable to reliably determine s/n.
