# Supplementary material for: Interfacing low-energy SAW nebulization with Liquid Chromatography-Mass Spectrometry for the analysis of biological samples
Source: Sci Rep. 2015 May 15;5:9736. doi: 10.1038/srep09736 (PMC4432867; doi:10.1038/srep09736)
Supplement: Supplementary Information — Supplementary figures 1 and 2 and table 1 [file srep09736-s1.pdf]

Supplementary Information

Interfacing low-energy SAW nebulization with Liquid Chromatography-Mass Spectrometry for the analysis of biological samples.

Karina Tveen-Jensen,[2‡] Frank Gesellchen,[1‡] Rab Wilson,[1‡] Corinne M. Spickett,[2] Jonathan M. Cooper[1] and Andrew R. Pitt[2]*

[1]Division of Biomedical Engineering, University of Glasgow, Oakfield Avenue, Glasgow, UK, G12 8LT

[2]School of Life and Health Sciences, Aston University, Aston Triangle, Birmingham, UK, B4 7ET.

*a.r.pitt@aston.ac.uk

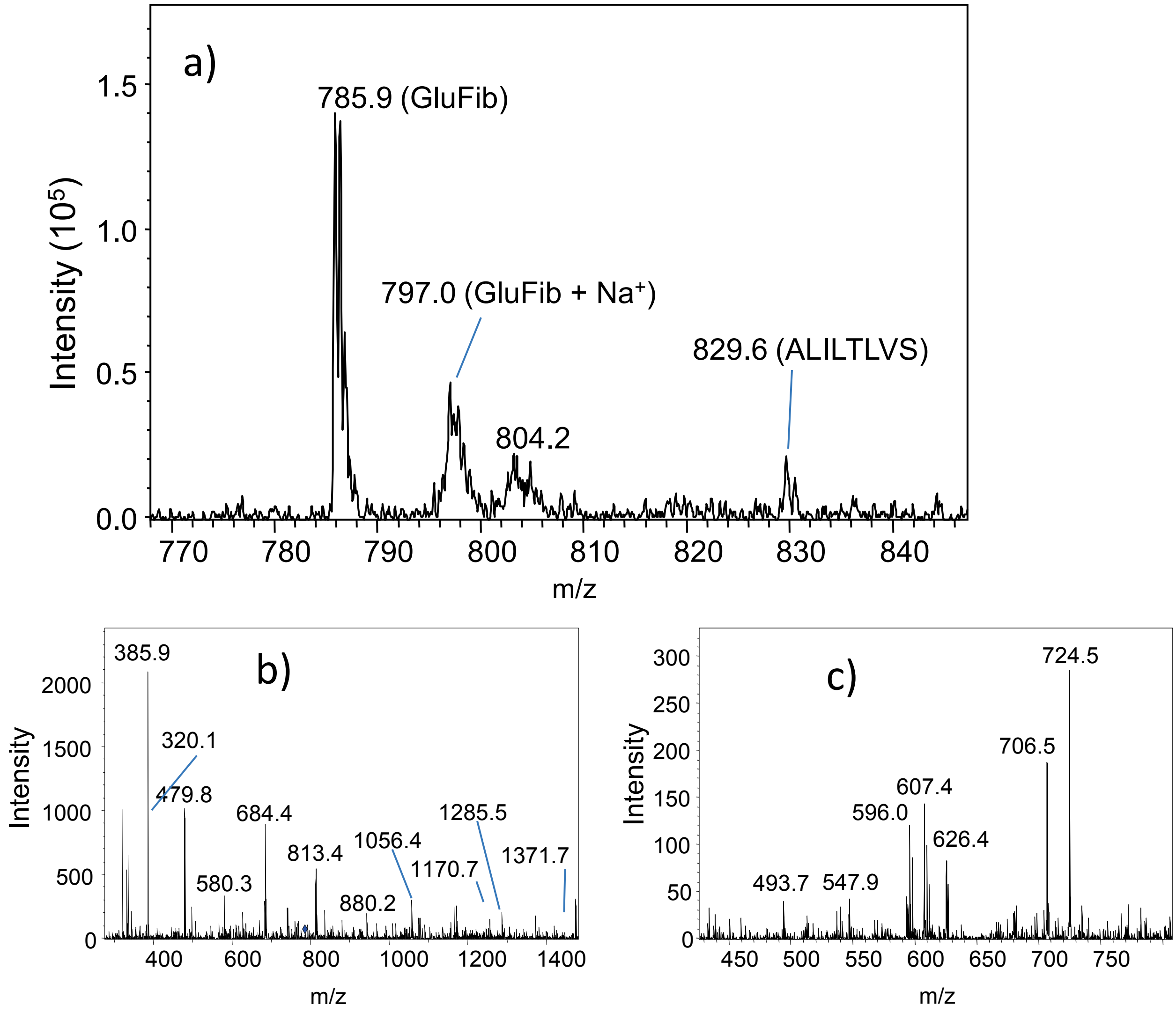

Supplementary Fig. 1. Data obtained from continuous infusion of a mixture of mixture of GluFib (2^+ ion, *m/z* 785.8 Da) and ALILTLVS (1^+ ion, *m/z* 829.6) in a 50:1 ratio using SAWN. a) MS spectrum of mixture, b) MSMS spectrum of *m/z* 785.9, and c) MSMS spectrum of *m/z* 829.6, confirming identity of peptide peaks. The peak at *m/z* 797.9 is the sodium adduct of GluFib. and *m/z* 804.2 is probably the potassium adduct.

SAWN 30 pmol

Protein View: ALBU_HUMAN

Serum albumin OS=Homo sapiens GN=ALB PE=1 SV=2

**Database:**	SwissProt
**Score:**	887
**Nominal mass (M_r):**	69321
**Calculated pI:**	5.92
**Taxonomy:**	Homo sapiens

Sequence similarity is available as an NCBI BLAST search of ALBU_HUMAN against nr.

Search parameters

**MS data file:**	DATA.TXT
**Enzyme:**	Trypsin: cuts C-term side of KR unless next residue is P.
**Variable modifications:**	Carbamidomethyl (C), Oxidation (M)

Protein sequence coverage: 50%

Matched peptides shown in ***bold red***.

```
  1 MKWVTFISLL FLFSSAYSRG VFRRDAHKSE VAHRFKDLGE ENFKALVLIA
 51 FAQYLQQCPF EDHVKLVNEV TEFAKTCVAD ESAENCDKSL HTLFGDKLCT
101 VATLRETYGE MADCCAKQEP ERNECFLQHK DDNPNLPRLV RPEVDVMCTA
151 FHDNEETFLK KYLYEIARRH PYFYAPELLF FAKRYKAAFT ECCQAADKAA
201 CLLPKLDELR DEGKASSAKQ RLKCASLQKF GERAFKAWAV ARLSQRFPKA
251 EFAEVSKLVT DLTKVHTECC HGDLLECADD RADLAKYICE NQDSISSKLK
301 ECCEKPLLEK SHCIAEVEND EMPADLPSLA ADFVESKDVC KNYAEAKDVF
351 LGMFLYEYAR RHPDYSVVLL LRLAKTYETT LEKCCAAADP HECYAKVFDE
401 FKPLVEEPQN LIKQNCELFE QLGEYKFQNA LLVRYTKKVP QVSTPTLVEV
451 SRNLGKVGSK CCKHPEAKRM PCAEDYLSVV LNQLCVLHEK TPVSDRVTKC
501 CTESLVNRRP CFSALEVDET YVPKEFNAET FTFHADICTL SEKERQIKKQ
551 TALVELVKHK PKATKEQLKA VMDDFAAFVE KCCKADDKET CFAEEGKKLV
601 AASQAALGL
```

SAWN 3 pmol

Protein View: ALBU_HUMAN

Serum albumin OS=Homo sapiens GN=ALB PE=1 SV=2

**Database:**	SwissProt
**Score:**	433
**Nominal mass (M_r):**	69321
**Calculated pI:**	5.92
**Taxonomy:**	Homo sapiens

Sequence similarity is available as an NCBI BLAST search of ALBU_HUMAN against nr.

Search parameters

**MS data file:**	DATA.TXT
**Enzyme:**	Trypsin: cuts C-term side of KR unless next residue is P.
**Variable modifications:**	Carbamidomethyl (C), Oxidation (M)

Protein sequence coverage: 42%

Matched peptides shown in ***bold red***.

```
  1 MKWVTFISLL FLFSSAYSRG VFRRDAHKSE VAHRFKDLGE ENFKALVLIA
 51 FAQYLQQCPF EDHVKLVNEV TEFAKTCVAD ESAENCDKSL HTLFGDKLCT
101 VATLRETYGE MADCCAKQEP ERNECFLQHK DDNPNLPRLV RPEVDVMCTA
151 FHDNEETFLK KYLYEIARRH PYFYAPELLF FAKRYKAAFT ECCQAADKAA
201 CLLPKLDELR DEGKASSAKQ RLKCASLQKF GERAFKAWAV ARLSQRFPKA
251 EFAEVSKLVT DLTKVHTECC HGDLLECADD RADLAKYICE NQDSISSKLK
301 ECCEKPLLEK SHCIAEVEND EMPADLPSLA ADFVESKDVC KNYAEAKDVF
351 LGMFLYEYAR RHPDYSVVLL LRLAKTYETT LEKCCAAADP HECYAKVFDE
401 FKPLVEEPQN LIKQNCELFE QLGEYKFQNA LLVRYTKKVP QVSTPTLVEV
451 SRNLGKVGSK CCKHPEAKRM PCAEDYLSVV LNQLCVLHEK TPVSDRVTKC
501 CTESLVNRRP CFSALEVDET YVPKEFNAET FTFHADICTL SEKERQIKKQ
551 TALVELVKHK PKATKEQLKA VMDDFAAFVE KCCKADDKET CFAEEGKKLV
601 AASQAALGL
```

ESI 3 pmol

Protein View: ALBU_HUMAN

Serum albumin OS=Homo sapiens GN=ALB PE=1 SV=2

**Database:**	SwissProt
**Score:**	417
**Nominal mass (M_r):**	69321
**Calculated pI:**	5.92
**Taxonomy:**	Homo sapiens

Sequence similarity is available as an NCBI BLAST search of ALBU_HUMAN against nr.

Search parameters

**MS data file:**	DATA.TXT
**Enzyme:**	Trypsin: cuts C-term side of KR unless next residue is P.
**Variable modifications:**	Carbamidomethyl (C), Oxidation (M)

Protein sequence coverage: 61%

Matched peptides shown in ***bold red***.

```
  1 MKWVTFISLL FLFSSAYSRG VFRRDAHKSE VAHRFKDLGE ENFKALVLIA
 51 FAQYLQQCPF EDHVKLVNEV TEFAKTCVAD ESAENCDKSL HTLFGDKLCT
101 VATLRETYGE MADCCAKQEP ERNECFLQHK DDNPNLPRLV RPEVDVMCTA
151 FHDNEETFLK KYLYEIARRH PYFYAPELLF FAKRYKAAFT ECCQAADKAA
201 CLLPKLDELR DEGKASSAKQ RLKCASLQKF GERAFKAWAV ARLSQRFPKA
251 EFAEVSKLVT DLTKVHTECC HGDLLECADD RADLAKYICE NQDSISSKLK
301 ECCEKPLLEK SHCIAEVEND EMPADLPSLA ADFVESKDVC KNYAEAKDVF
351 LGMFLYEYAR RHPDYSVVLL LRLAKTYETT LEKCCAAADP HECYAKVFDE
401 FKPLVEEPQN LIKQNCELFE QLGEYKFQNA LLVRYTKKVP QVSTPTLVEV
451 SRNLGKVGSK CCKHPEAKRM PCAEDYLSVV LNQLCVLHEK TPVSDRVTKC
501 CTESLVNRRP CFSALEVDET YVPKEFNAET FTFHADICTL SEKERQIKKQ
551 TALVELVKHK PKATKEQLKA VMDDFAAFVE KCCKADDKET CFAEEGKKLV
601 AASQAALGL
```

Supplementary fig. 2. Details of results of MASCOT search showing scores and sequence coverage. Full details of peptide identifications are presented in supplementary table 1.

Supplementary table 1. Full peptide data for MASCOT searches.

SAWN 30 pmoles digest on colum

Serum albumin OS=Homo sapiens GN=ALB PE=1 SV=2 ALBU_HUMAN
Digest Matches (Score: 886.77)
Modifications: Global: Optional: Carbamidomethyl (C), Oxidation (M),
Search Parameter: Charge=2+ and 3+, MS Tol.:0.800000 Da, MSMS Tol.:0.500000 Da, Trypsin , Mascot 2.4.1, SwissProt SwissProt_2013_08.fasta

Measured *m/z* (Da)	Calculated MH+ (Da)	Measured Mr (Da)	Calculated Mr (Da)	Deviation (Da)	Deviation (ppm)	MascotScore	Retention time (min)	Protein Range	Sequence
480.7	959.55	959.39	959.56	-0.16	-170.9	22	28.11	427 - 434	FQNALLVR
507.13	1012.59	1012.25	1012.59	-0.34	-335.8	52	33.51	599 - 609	LVAASQAALGL
575.11	1148.61	1148.21	1148.61	-0.4	-350.41	35	27.29	66 - 75	LVNEVTEFAK
575.29	1148.61	1148.57	1148.61	-0.04	-32.28	42	27.96	66 - 75	LVNEVTEFAK
613.78	1225.6	1225.54	1225.6	-0.06	-47.68	50	23.5	35 - 44	FKDLGEENFK
672.21	1341.63	1342.4	1341.63	0.77	573.01	70	35.89	570 - 581	AVMDDFAAFVEK
756.36	1510.83	1510.7	1510.84	-0.13	-88.74	46	29.32	439 - 452	VPQVSTPTLVEVSR
774.89	1547.67	1547.76	1547.67	0.08	54.16	53	23.95	185 - 198	YKAAFTECCQAADK
812.43	1622.78	1622.84	1622.78	0.06	36.09	45	45.45	348 - 360	DVFLGMFLYEYAR
812.55	1622.78	1623.08	1622.78	0.3	185.09	46	44.52	348 - 360	DVFLGMFLYEYAR
547.21	1638.93	1638.61	1638.93	-0.32	-197.02	34	26.74	438 - 452	KVPQVSTPTLVEVSR
820.41	1638.93	1638.8	1638.93	-0.13	-81.41	57	26.4	438 - 452	KVPQVSTPTLVEVSR
829.31	1656.74	1656.6	1656.75	-0.15	-89.27	72	32.35	414 - 426	QNCELFEQLGEYK
829.35	1656.74	1656.69	1656.75	-0.05	-31.81	69	32.08	414 - 426	QNCELFEQLGEYK
920.99	1839.91	1839.97	1839.91	0.07	35.43	64	36.95	566 - 581	EQLKAVMDDFAAFVEK
937.99	1874.01	1873.96	1874.01	-0.05	-25.7	52	31.19	89 - 105	SLHTLFGDKLCTVATLR
949.95	1897.99	1897.88	1897.99	-0.11	-55.35	53	34.52	169 - 183	RHPYFYAPELLFFAK
633.68	1897.99	1898.01	1897.99	0.02	11.68	45	34.44	169 - 183	RHPYFYAPELLFFAK
955.88	1909.92	1909.74	1909.92	-0.18	-94.74	51	29.63	509 - 524	RPCFSALEVDETYVPK

966.46	1931.03	1930.9	1931.03	-0.13	-66.51	77	30.77	89 - 105	SLHTLFGDKLCTVATLR
966.49	1931.03	1930.96	1931.03	-0.07	-36.37	74	30.09	89 - 105	SLHTLFGDKLCTVATLR
977.47	1952.92	1952.92	1952.92	0.01	3.72	63	33.79	187 - 205	AAFTECCQAADKAACLLPK
1005.9	2009.94	2009.79	2009.94	-0.15	-73.94	34	31.88	187 - 205	AAFTECCQAADKAACLLPK
1005.9	2009.94	2009.79	2009.94	-0.15	-73.94	33	31.88	187 - 205	AAFTECCQAADKAACLLPK
1022.96	2044.09	2043.91	2044.09	-0.18	-87	53	34.16	397 - 413	VFDEFKPLVEEPQNLIK
1023.05	2044.09	2044.08	2044.09	-0.01	-3.83	36	34.96	397 - 413	VFDEFKPLVEEPQNLIK
682.39	2044.09	2044.16	2044.09	0.07	35.86	40	34.14	397 - 413	VFDEFKPLVEEPQNLIK
708.93	2123.98	2123.76	2123.98	-0.22	-103.21	45	29.12	187 - 205	AAFTECCQAADKAACLLPK
749.05	2244.07	2244.13	2244.07	0.06	26.78	22	32.52	185 - 205	YKAAFTECCQAADKAACLLPK
1150.45	2299.1	2298.88	2299.1	-0.21	-92.84	61	39.7	342 - 360	NYAEAKDVFLGMFLYEYAR
806.06	2415.14	2415.16	2415.14	0.02	7.29	27	28.24	185 - 205	YKAAFTECCQAADKAACLLPK
843.31	2527.09	2526.91	2527.09	-0.18	-71.79	25	23.81	265 - 286	VHTECCHGDLLECADDRADLAK
843.31	2527.09	2526.91	2527.09	-0.18	-71.79	25	23.81	265 - 286	VHTECCHGDLLECADDRADLAK
843.31	2527.09	2526.91	2527.09	-0.18	-71.79	6	23.81	265 - 286	VHTECCHGDLLECADDRADLAK
843.39	2527.09	2527.16	2527.09	0.07	27.81	26	24.5	265 - 286	VHTECCHGDLLECADDRADLAK
843.39	2527.09	2527.16	2527.09	0.07	27.81	2	24.5	265 - 286	VHTECCHGDLLECADDRADLAK
843.39	2527.09	2527.16	2527.09	0.07	27.81	2	24.5	265 - 286	VHTECCHGDLLECADDRADLAK
862.37	2584.11	2584.08	2584.11	-0.03	-13.15	12	23.04	265 - 286	VHTECCHGDLLECADDRADLAK
867.13	2598.29	2598.38	2598.29	0.09	33.19	43	36.25	414 - 434	QNCELFEQLGEYKFQNALLVR
879.43	2635.22	2635.26	2635.22	0.04	13.95	18	22.18	118 - 138	QEPERNECFLQHKDDNPNLPR

ESI 3 pmoles digest on column

Global peptide results

Serum albumin OS=Homo sapiens GN=ALB PE=1 SV=2 ALBU_HUMAN

Digest Matches (Score: 416.80)

Modifications: Global: Optional: Carbamidomethyl (C), Oxidation (M),

Search Parameter: Charge=2+ and 3+, MS Tol.:1.000000 Da, MSMS Tol.:0.500000 Da, Trypsin , Mascot 2.4.1, SwissProt SwissProt_2013_08.fasta

Meas. M/z	Calc. MH+	Meas. Mr	Calc. Mr	Dev.(Da)	Dev.(ppm)	MascotSco	Rt(min)	Range	Sequence
463.85	926.49	925.68	926.49	-0.8	-868.1	15	25.85	162 - 168	YLYEIAR

480.31	959.55	958.61	959.56	-0.94	-980.03	24	26.44	427 - 434	FQNALLVR
500.83	999.6	999.65	999.6	0.05	50.43	18	27.79	550 - 558	QTALVELVK
575.1	1148.61	1148.19	1148.61	-0.42	-365.03	36	26.85	66 - 75	LVNEVTEFAK
575.13	1148.61	1148.25	1148.61	-0.36	-310.53	40	26.27	66 - 75	LVNEVTEFAK
613.7	1225.6	1225.39	1225.6	-0.21	-172.35	36	22.48	35 - 44	FKDLGEENFK
629.23	1256.52	1256.44	1256.52	-0.07	-58.65	34	23.46	187 - 198	AAFTECCQAADK
671.78	1341.63	1341.55	1341.63	-0.07	-55.48	47	36.51	570 - 581	AVMDDFAAFVEK
671.85	1341.63	1341.68	1341.63	0.06	42.31	26	35.94	570 - 581	AVMDDFAAFVEK
734.36	1466.84	1466.72	1466.84	-0.12	-82.16	28	28.41	361 - 372	RHPDYSVVLLLR
756.35	1510.83	1510.69	1510.84	-0.15	-97.61	55	28.23	439 - 452	VPQVSTPTLVEVSR
774.76	1547.67	1547.5	1547.67	-0.17	-109.82	39	22.69	185 - 198	YKAAFTECCQAADK
812.37	1622.78	1622.73	1622.78	-0.05	-28.86	40	45.29	348 - 360	DVFLGMFLYEYAR
812.51	1622.78	1623	1622.78	0.22	137.52	29	44.61	348 - 360	DVFLGMFLYEYAR
547.01	1638.93	1638	1638.93	-0.93	-566.23	25	25.5	438 - 452	KVPQVSTPTLVEVSR
820.38	1638.93	1638.75	1638.93	-0.18	-112.53	26	25.72	438 - 452	KVPQVSTPTLVEVSR
820.38	1638.77	1638.75	1638.78	-0.02	-14.5	39	41.89	348 - 360	DVFLGMFLYEYAR
825.92	1649.89	1649.83	1649.89	-0.06	-36.69	69	35.68	250 - 264	AEFAEVSKLVTDLTK
928.89	1855.9	1855.77	1855.9	-0.13	-72.17	36	37.51	566 - 581	EQLKAVMDDFAAFVEK
633.59	1897.99	1897.75	1897.99	-0.24	-125.52	48	34.46	169 - 183	RHPYFYAPELLFFAK
637.56	1909.92	1909.66	1909.92	-0.27	-139.55	34	28.83	509 - 524	RPCFSALEVDETYVPK
977.39	1952.92	1952.77	1952.92	-0.15	-75.96	22	33.52	187 - 205	AAFTECCQAADKAACLLPK
666.22	1995.92	1995.65	1995.92	-0.27	-137.14	28	21.62	123 - 138	NECFLQHKDDNPNLPR
682.3	2044.09	2043.86	2044.09	-0.22	-109.73	53	35.04	397 - 413	VFDEFKPLVEEPQNLIK
689.92	2066.96	2066.74	2066.96	-0.22	-106.56	14	28.75	187 - 205	AAFTECCQAADKAACLLPK
753.96	2259.01	2258.87	2259.02	-0.14	-63.49	42	33.02	525 - 543	EFNAETFTFHADICTLSEK
767.35	2299.1	2299.02	2299.1	-0.08	-34.29	20	40.09	342 - 360	NYAEAKDVFLGMFLYEYAR
783.58	2348.03	2347.72	2348.03	-0.31	-130.33	63	28.97	98 - 117	LCTVATLRETYGEMADCCAK
843.29	2527.09	2526.85	2527.09	-0.24	-93.63	12	22.96	265 - 286	VHTECCHGDLLECADDRADLAK
865.06	2592.23	2592.16	2592.24	-0.08	-29.96	50	33.31	139 - 160	LVRPEVDVMCTAFHDNEETFLK
892.11	2673.31	2673.32	2673.31	0.01	3.89	17	39.71	469 - 490	RMPCAEDYLSVVLNQLCVLHEK
926.74	2777.35	2777.19	2777.35	-0.16	-56.89	18	29.79	139 - 161	LVRPEVDVMCTAFHDNEETFLKK

SAWN 3 pmoles digest on column

Global peptide results
Serum albumin OS=Homo sapiens GN=ALB PE=1 SV=2 ALBU_HUMAN
Digest Matches (Score: 432.53)
Modifications: Global: Optional: Carbamidomethyl (C), Oxidation (M),
Search Parameter: Charge=2+ and 3+, MS Tol.:1.000000 Da, MSMS Tol.:0.500000 Da, Trypsin , Mascot 2.4.1, SwissProt SwissProt_2013_08.fasta

Meas. M/z	Calc. MH+	Meas. Mr	Calc. Mr	Dev.(Da)	Dev.(ppm)	MascotSco	Rt(min)	Range	Sequence
464.24	926.49	926.47	926.49	-0.02	-16.28	9	27.59	162 - 168	YLYEIAR
500.75	999.6	999.48	999.6	-0.12	-120.44	14	29.23	550 - 558	QTALVELVK
565.26	1127.69	1128.51	1127.69	0.82	723.48	46	25.4	549 - 558	KQTALVELVK
575.31	1148.61	1148.61	1148.61	0	-0.42	30	28.22	66 - 75	LVNEVTEFAK
672.17	1341.63	1342.32	1341.63	0.69	513.82	63	36.39	570 - 581	AVMDDFAAFVEK
693.89	1385.61	1385.77	1385.61	0.15	109.97	37	23.02	287 - 298	YICENQDSISSK
716.99	1431.75	1431.97	1431.75	0.23	157.95	8	21.78	299 - 310	LKECCEKPLLEK
717.02	1431.75	1432.03	1431.75	0.28	195.11	35	21.35	299 - 310	LKECCEKPLLEK
722.36	1442.63	1442.71	1442.63	0.07	48.88	18	21.89	287 - 298	YICENQDSISSK
734.37	1466.84	1466.72	1466.84	-0.11	-75.89	47	29.38	361 - 372	RHPDYSVVLLLR
745.39	1488.77	1488.76	1488.77	-0.01	-5.86	43	20.67	299 - 310	LKECCEKPLLEK
745.39	1488.77	1488.76	1488.77	-0.01	-5.86	34	20.67	299 - 310	LKECCEKPLLEK
756.37	1510.83	1510.72	1510.84	-0.12	-79.08	39	29.36	439 - 452	VPQVSTPTLVEVSR
820.4	1638.93	1638.78	1638.93	-0.15	-91.79	50	26.47	438 - 452	KVPQVSTPTLVEVSR
820.47	1638.93	1638.93	1638.93	0	-2.95	50	27.26	438 - 452	KVPQVSTPTLVEVSR
829.32	1656.74	1656.63	1656.75	-0.12	-72.13	40	32.41	414 - 426	QNCELFEQLGEYK
871.9	1741.89	1741.79	1741.89	-0.1	-57.84	23	38.08	170 - 183	HPYFYAPELLFFAK
937.98	1874.01	1873.95	1874.01	-0.06	-33.7	44	31.62	89 - 105	SLHTLFGDKLCTVATLR
949.96	1897.99	1897.9	1897.99	-0.09	-45.44	43	34.9	169 - 183	RHPYFYAPELLFFAK
955.89	1909.92	1909.77	1909.92	-0.15	-80.39	34	29.93	509 - 524	RPCFSALEVDETYVPK
966.47	1931.03	1930.93	1931.03	-0.1	-50.45	61	30.46	89 - 105	SLHTLFGDKLCTVATLR
1022.96	2044.09	2043.9	2044.09	-0.18	-89.83	44	34.58	397 - 413	VFDEFKPLVEEPQNLIK
830.72	2489.28	2489.14	2489.28	-0.14	-55.93	61	41.46	45 - 65	ALVLIAFAQYLQQCPFEDHVK

862.3	2584.11	2583.87	2584.11	-0.24	-94.65	12	23.6	265 - 286	VHTECCHGDLLECADDRADLAK
892.41	2673.31	2674.2	2673.31	0.89	334.38	17	39.48	469 - 490	RMPCAEDYLSVVLNQLCVLHEK
907.74	2720.33	2720.21	2720.33	-0.12	-44.45	39	31.8	139 - 161	LVRPEVDVMCTAFHDNEETFLKK
